# Distinguishing between Isobaric Ions Using Microdroplet Hydrogen–Deuterium Exchange Mass Spectrometry

**DOI:** 10.3390/metabo11110728

**Published:** 2021-10-23

**Authors:** Xiaowei Song, Jia Li, Mohammad Mofidfar, Richard N. Zare

**Affiliations:** 1 Department of Chemistry, Stanford University, Stanford, CA 94305, USA; songxw@stanford.edu (X.S.); mxm801@stanford.edu (M.M.); 2 Department of Chemistry, Fudan University, Shanghai 200438, China; 18110220076@fudan.edu.cn

**Keywords:** hydrogen-deuterium exchange, isobaric ions, ambient ionization mass spectrometry, microdroplets

## Abstract

Isobaric ions having the same mass-to-charge ratio cannot be separately identified by mass spectrometry (MS) alone, but this limitation can be overcome by using hydrogen–deuterium exchange (HDX) in microdroplets. Because isobaric ions may contain a varied number of exchangeable sites and different types of functional groups, each one produces a unique MS spectral pattern after droplet spray HDX without the need for MS/MS experiments or introduction of ion mobility measurements. As an example of the power of this approach, isobaric ions in urinary metabolic profiles are identified and used to distinguish between healthy individuals and those having bladder cancer.

## 1. Introduction

Ambient ionization mass spectrometry (AIMS) refers to the strategy that directly analyzes the sample’s composition or target species under atmospheric and room-temperature conditions [1,2]. AIMS can successfully detect a wide range of chemical species, such as synthetic drugs; pesticides; and endogenous metabolites, including amino acids, fatty acids, nucleosides, carboxylic acids, carbohydrates, aldehydes, glycerophospholipids, etc. [3,4,5,6,7]. AIMS has advantages in that it is free from labor-intensive pretreatment and thus can be very useful for those on-site detection scenarios that demand quick feedback about the test result, such as forensic detection of controlled drugs [8,9,10], and point-of-care medical emergencies. In the past, AIMS has been used to acquire the metabolic profile for different biological fluids, such as urine, saliva, serum, and extracellular vesicles [8,11,12,13], which are found in breast cancer, prostate cancer, cervical cancer, oral cancer, etc. [12,13,14,15,16].

However, AIMS has its own limitations. Unlike the combination of chromatographic separation with mass spectrometric detection, which separates most components before detection, this direct infusion mode gains the convenience of directly characterizing the sample’s profile at the cost of losing in-depth molecular resolving ability. Specifically, AIMS fails to distinguish those species that have the same molecular weight and formula (isobaric ions). Consequently, it is difficult to assign the unambiguous identity to a certain ion without further investigating and matching the MS/MS pattern. These issues pose a methodological challenge to direct infusion-based metabolomic studies.

Ion-mobility mass spectrometry (IMS) provides an alternative possibility of distinguishing isobaric ions based on their different collision cross-sections. Not surprisingly, IMS can more easily achieve good performance on macromolecules (e.g., peptides and proteins) or small molecules that have obvious differences in molecular shape, size, or spatial conformation [17]. For isomers that have very intricate structural differences, the current IMS still has a very limited ability to achieve an ideal separation. To complement IMS, several reports introduced deuterium reagents into the mobility cell to conduct gas-phase hydrogen–deuterium exchange (GP-HDX) [18]. Compared to IMS, GP-HDX helps to probe more intricate structural details, such as the number of exchangeable proton sites from proteins, carbohydrates, amines, lipids, etc. [19,20,21,22] Practically, this IMS/GP-HDX combination needs a specially modified IMS setup and consumes more deuterium reagent. In recent years, the Valentine group has conducted a series of systematic HDX studies on small molecules. This work ranges from predicting the HDX pattern of functional groups to the proof-of-concept droplet HDX study for metabolomic studies [18,20,23]. Motivated by this past work, we were inspired to integrate liquid-phase HDX with AIMS to develop a simple, robust, and cost-effective method for distinguishing isobaric ions in untargeted metabolomic studies.

**Figure 1:**
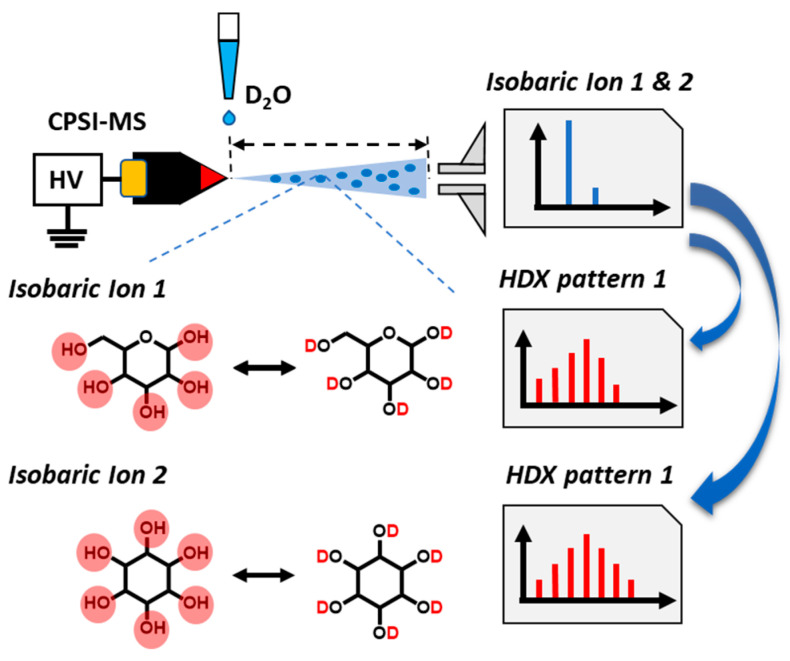
Workflow for using HDX–CPSI-MS for distinguishing between isobaric ions.

Previously, our group has developed a series of polymer-based ambient ionization methods [24,25,26,27], which have shown advantages in weak absorption of hydrophilic species, stronger ion intensity, and more stable signal duration [28]. Among these AIMS methods, conductive-polymer-spray-ionization mass spectrometry (CPSI-MS) has been successfully used for salivary metabolic profiling and oral cancer diagnosis [12]. Only a few microliters of methanol–water solvent suffice to desorb and ionize a wide range of metabolites within a few seconds. In other studies, we have also shown that the transient process of microdroplet HDX can be well captured by a DESI-MS system [29]. In this study, a proportion of methanol–deuterium water is used as the desorption solvent in CPSI-MS. When the deuterium-containing solvent contacts a dried sample spot on the conductive polymer tip with a high voltage applied, the microdroplet HDX process commences and the post-HDX metabolic profile can be easily recorded. The general workflow is schematically illustrated in Figure 1.

## 2. Results and Discussion

### 2.1. Rapid Recognition of Opioid Narcotics

**Figure 2:**
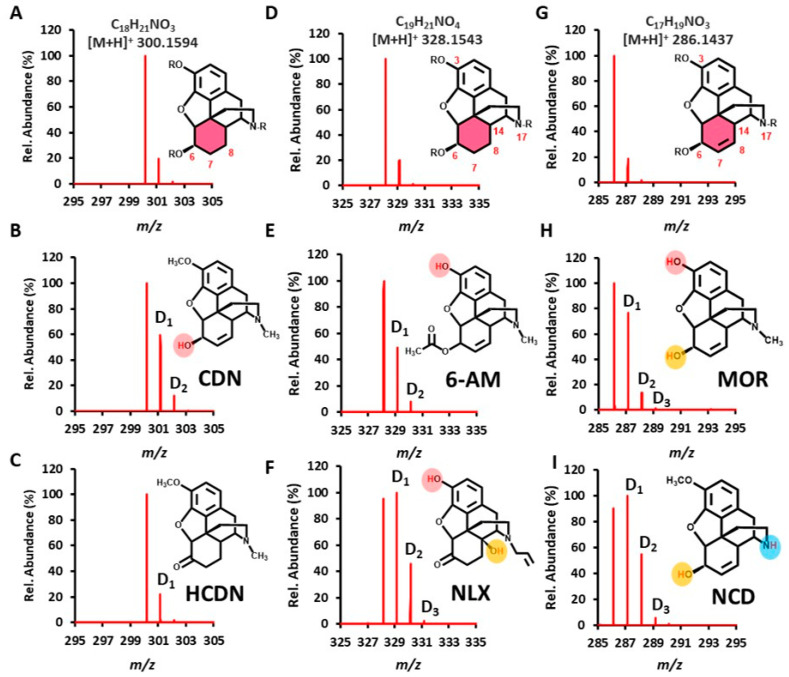
HDX pattern for distinguishing between three pairs of opioid drug ions. (**A**,**D**,**G**) High-resolution mass spectra and parent structures for three pairs of isobaric ions in mixed solutions: (**A**) C_18_HNO_3_, (**D**) C_19_H_21_NO_4_, and (**G**) C_17_H_19_NO_3_. Post-HDX mass spectra for (**B**) codeine (CDN), (**C**) hydrocodone (HCDN), (**E**) 6-acetyl morphine (6-AM), (**F**) naloxone (NLX), (**H**) morphine (MOR), and (**I**) norcodeine (NCD). Only deuterium peaks are annotated in mass spectra according to the mass shift of one deuteration, which is around 1.0063 ± 0.0002. Please note that the resolution of the mass spectrometer being used is unable to distinguish between the shift from ^2^H and those from ^13^C and ^15^N.

We first selected several pairs of frequently abused opioid narcotics as model compounds for testing the feasibility of microdroplet HDX combined with CPSI-MS in distinguishing isobaric ions. The first pair of opioids are codeine and hydrocodone, which have the same formula (C_18_H_21_NO_3_) and an *m*/*z* value ([M+H]^+^, 300.1594, Figure 2A). However, when focusing on precise structural differences, codeine has one active proton in the 6-hydroxyl group in contrast to hydrocodone that has only carbonyl and no active proton in the carbon-6 position. Therefore, these two compounds can be very easily distinguished in the HDX-CPSI mass spectra from their differences in deuterium peak number (Figure 2B,C). By contrast, it is relatively difficult to distinguish these two compounds from examining the MS/MS spectra which have quite similar fragment ion patterns (Supplementary Materials Figure S1) under the same CID energy (30 V). It should be noted that a protonated ion derived from the compound with no exchangeable proton could also yield one deuterated peak under HDX–CPSI-MS analysis because of the deuterium cation dissociated from heavy water. The deuterium peak can be easily recognized from the native isotope caused by its specific mass shift around 1.0063, measured by the high-resolution mass spectrometer. Unfortunately, a deuterium peak [(M-H+D)+H]^+^ will cover peaks arising from native isotopes (^13^C, ^2^H, and ^15^N) inside the peak profile given the present mass resolution of 120,000 and full-width at half maximum (FWHM) around 0.002. Therefore, in this situation, the increase of the isotope peak intensity is mainly used for judging the HDX process.

The second pair of isobaric compounds are 6-acetylmorphine and naloxone (C_19_H_21_NO4, [M+H]^+^ 328.1543, Figure 2D). Apart from one phenolic hydroxyl group that they both have in the 3-position, naloxone carries an extra hydroxyl group in the 14-position. Thus, it can yield one more deuterium peak than 6-acetylmorphine (Figure 2E,F). In this case, the D_1_ peak of naloxone also becomes the base peak instead of D_0_. This behavior has a simple explanation. Either one of two exchangeable proton sites from naloxone contributed to the D_1_ peak. For another more important reason, the hydroxyl group has a faster HDX rate compared to the phenol group [30].

The second study case raises the question of whether microdroplet HDX can also distinguish isobaric ions that have the same number of exchangeable proton sites from different functional groups. Thus, we investigated the third pair of opioid compounds, morphine and norcodeine, which have the same formula of C_17_H_19_NO_3_ and protonated ion at *m*/*z* 286.1437 (Figure 2G). They both have a hydroxyl group in the 6-position, but morphine possesses one phenol group in the 3-position whereas norcodeine has one imine group in the 17-position. As a result, the D_1_ and D_2_ from norcodeine were greatly increased compared to that from morphine, although there were three deuterium peaks for both of them (Figure 2H,I). Norcodeine’s base peak became D_1_, but morphine’s base peak was still D_0_, involving the exchange rate difference between phenol and imine protons when the pH ranged from 4.0 to 10.0, which is predominantly catalyzed by the base. The liquid-phase back exchange difference may also play a role in distinguishing these two isobaric ions, which we present in the following section. This result indicates that HDX-based isobaric ions can be distinguished according to not only the exchangeable proton number but also the functional groups they possess.

### 2.2. Distinguishing Isobaric Ions That Are Challenging to Tell Apart by MS/MS

**Figure 3:**
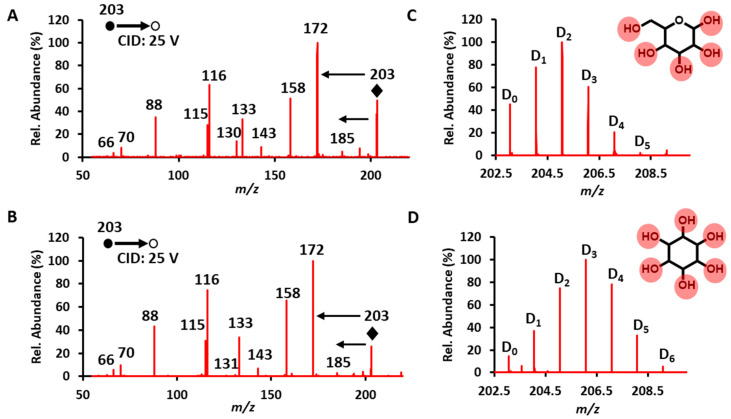
HDX for isobaric ions that also share similar MS2 patterns. CID-MS/MS spectra for (**A**) glucose and (**B**) inositol; post-HDX, high-resolution mass spectra for (**C**) glucose and (**D**) inositol. The diamonds denote the precursor ions, and circles in the diagrams identify exchangeable sites.

Although MS/MS dissociation still serves as the major strategy to identify the above-mentioned compounds from their fragmentation patterns (Supplementary Materials Figures S1–S3), microdroplet HDX provides an alternative method for simple and quick isobaric ion recognition. Distinguishing between isobaric ions based on different deuterium isotope intensity patterns should be regarded as similar to distinguish them based on the different intensities of their product ions if previously well characterized (Supplementary Materials Figures S1–S3). To give a better demonstration, glucose and inositol were selected as typical cases. They both exist in all varieties of biological fluids (e.g., serum, saliva, urine, etc.) and act as carbon and energy sources to maintain body functions. However, these two types of metabolites were difficult to be discerned from the metabolic profile. We also investigated their MS/MS fragmentation patterns under CID (energy 25 V). As shown in Figure 3A,B, MS/MS experiments failed to differentiate these two metabolites based on the top 10 fragment ions. Their patterns were completely the same, owing to the similarities in structure and functional groups. Fortunately, from the HDX mass spectrum, there is one more deuterium peak (D_6_) in the inositol than that in glucose. Moreover, the D_3_ becomes the base peak instead of D_2_ in glucose (Figure 3C,D). Thus, glucose and inositol can be readily distinguished by microdroplet HDX/mass spectrometry. We presented this example to illustrate in these situations that the MS/MS patterns are quite close with each other; microdroplet HDX is a much simpler but effective strategy if possible isobaric ions happen to have a different number of exchangeable protons. Using the similar strategy, we made a careful retrospective analysis on results of our previous studies on oral cancer, and we summarize a list of isobaric ions that can be frequently detected from the CPSI-MS-based saliva and serum-based metabolic profiles for reference (Supplementary Materials Table S1).

### 2.3. Correcting Falsely Matched Adduct Ions

**Figure 4:**
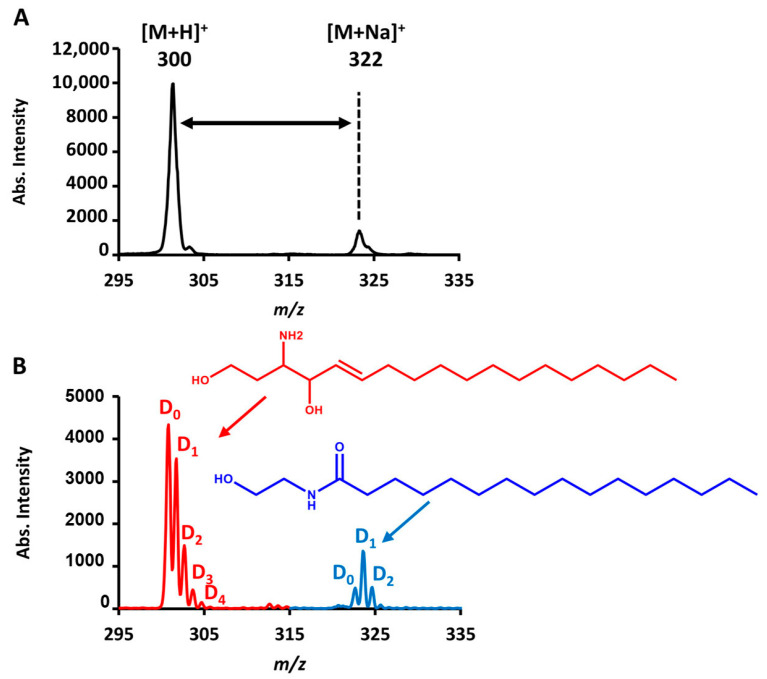
HDX–CPSI-MS for adduct ion differentiation. (**A**) Mass spectra of a pair of protonated and sodiated ions recorded by an LTQ mass spectrometer and (**B**) the distinguishing result for the pair of adduct ions, which are sphingosine (red, *m*/*z* 300, [M+H]^+^) and palmitoylethanolamide (blue, *m*/*z* 322, [M+Na]^+^).

In the untargeted metabolic profiling by AIMS, the delta *m*/*z* shift often helps to indicate the type of adduct ion. However, this strategy sometimes causes a misleading judgment. We found a very interesting case from the ongoing study about serum metabolomics for oral cancer. The suspect ions were located at *m*/*z* 300.2897 and 322.2716, which may be normally assigned to a metabolite with one proton or with one sodium adduct (Figure 4A), respectively. However, when we retested a self-collected dried saliva spot sample by HDX-CPSI-MS (LTQ), it was surprisingly seen that the ion at *m*/*z* 300 yielded four deuterium peaks whereas the ion at *m*/*z* 322 generated two deuterium peaks (Figure 4B). After searching the human metabolome database (HMDB), we narrowed down our attention to the two most possible metabolites, namely palmitoylethanolamide, and sphingosine. The former one has four exchangeable proton sites and the latter one has only two exchangeable proton sites. In this case, the pair of [M+H]^+^ and [M+Na]^+^ adduct ions seemed to derive from the same metabolite but actually not. This is probably because of their affinity differences to positively charged species. This study case illustrates that microdroplet HDX can help to reduce the chance of a false match of adduct ion pairs in AIMS-based metabolic profiling.

### 2.4. Comparison with Gas-Phase HDX

**Figure 5:**
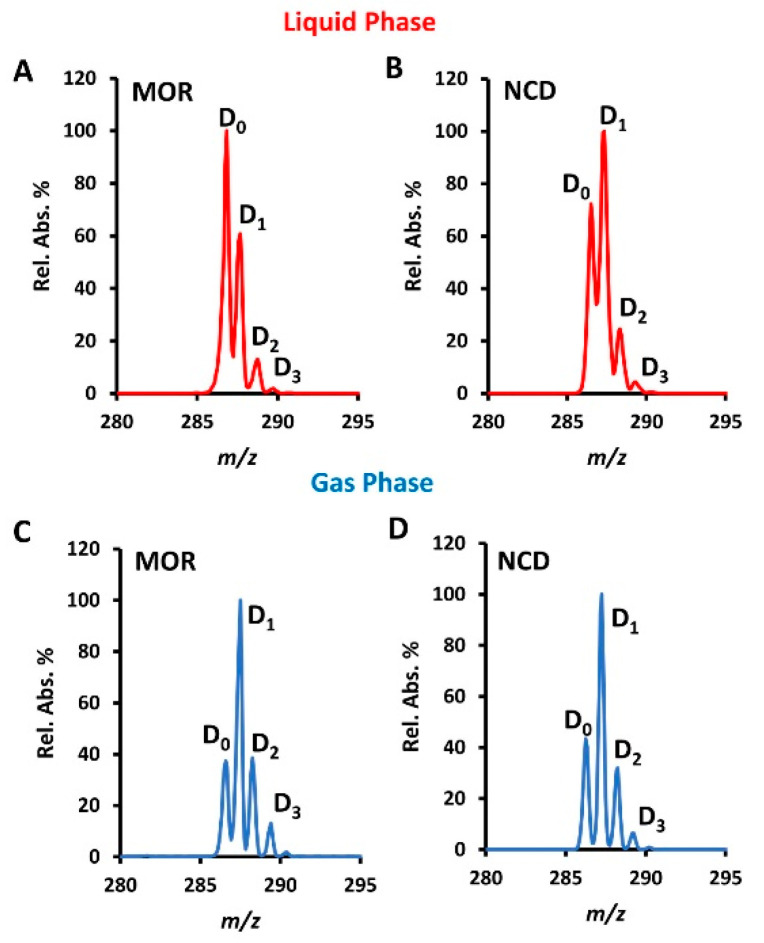
Comparison of HDX that happens in the liquid phase and the gas phase. MOR represents morphine and NCD represents norcodeine. Mass spectra for (**A**) morphine (MOR), (**B**) norcodeine (NCD) after conducting liquid-phase HDX, (**C**) MOR, and (**D**) NCD after conducting gas-phase HDX.

The HDX process we implemented by the CPSI method is mainly happening in charged microdroplets during the travel between the conductive polymer tip and the MS inlet on the microsecond timescale. In contrast to the gas-phase HDX that uses deuterium reagents in the gas phase, the back exchange also exists in this liquid-phase HDX process [31,32]. However, from the point of qualitative analysis, this insufficient H/D exchange becomes an advantage for isobaric ion discrimination. Taking morphine and norcodeine as examples, we compared patterns of the two compounds’ HDX, which happened under liquid-phase and gas-phase conditions. The apparently different patterns (Figure 5A,B) observed from the previous study become consistent with each other during gas-phase HDX (Figure 5C,D). This is largely because the much faster exchange rate in the gas phase without back exchange eliminates the functional-group-dependent HDX difference in the liquid phase. In this regard, insufficient HDX caused by back exchange in the liquid phase provides more detailed information on isobaric ion structure.

### 2.5. Creating New Dimension and Features for Metabolic Profiling

**Figure 6:**
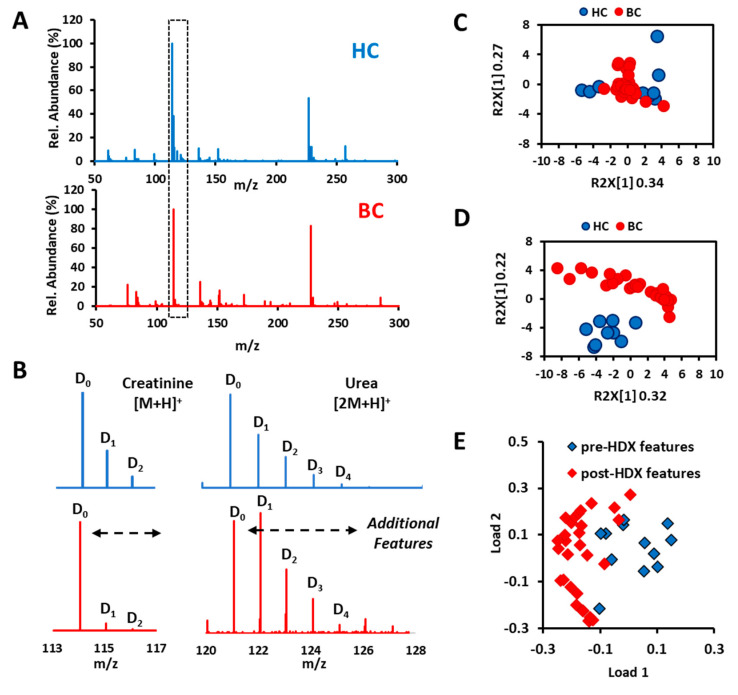
HDX for statistics-free highlight of the cancer signature. (**A**) Average mass spectra with HDX acquired from bladder cancer and normal contrast urine samples. (**B**) Typical metabolite ions that have a significant difference between two groups can be directly observed. (**C**) PCA score plots based on the original features collected from the plain metabolic profile. (**D**) PCA score plots based on the original features and newly generated deuterium peaks as the additional features. (**E**) PCA loading plot that visualizes the weight coefficient of each feature to the clustering. The dashed region in (**A**) is enlarged to form what is shown in (**B**).

After illustrating the usage of microdroplet HDX on isobaric ion recognition, we continued investigating its practical value in AIMS-based untargeted metabolic profiling. There were 30 bladder cancer (BC) and nine healthy control (HC) urine samples collected for this proof-of-concept study. The post-HDX mass spectra from BC and HC samples were averaged for comparison. Most metabolites were mainly distributed within the range of *m*/*z* 50–300 under positive scan mode (Figure 6A). The deuteration peaks can be easily discerned based on the specific mass shift around 1.0063. Then we carefully checked through the mass spectra and selected the top 10 ions whose peaks have the most deuteration shifts. Urea ([2M+H]^+^) and creatinine ([M+H]^+^) were the most typical ones because their isotope peak intensity differences between the two groups were so obvious to be directly read out from the average mass spectra (Figure 6B). It is probably originated from the different pH environments [29].

Because there were more HDX-generated deuterated peaks in the metabolic profiles compared to the pre-HDX metabolic profile, we evaluated the performance of principal component analysis (PCA) on sample clustering according to the native and HDX metabolic profiles, respectively. In the contrast, the top 10 ions from pre-HDX metabolic profiles were selected as original features. For HDX metabolic profile, an extra 31 deuterium ions were selected as the newly created features apart from the original top 10 featured ions. As a result, we can clearly see from the score plots that PCA, as an unsupervised machine learning method, failed to separate cleanly the BC from HC merely based on the top 12 ions (Figure 6C) but performed well when taking the corresponding 29 deuterium ions into account (Figure 6D). These results clearly demonstrated that HDX–CPSI-MS helps to create a new dimensional feature (number of exchangeable proton sites) to enhance the profile difference between two groups for pattern recognition. From the loading plots in Figure 6E, we learned that these newly created deuterium peaks indeed make contributions to the sample grouping.

### 2.6. Relative and Absolute Quantitation

Often in AIMS-based metabolic profiling more than one overlapped isobaric ion is present. This situation poses challenges for not only qualitative differentiation but also the quantitative estimation of each species. Consequently, we were motivated to carry out an absolute quantitation study on three pairs of mixed opioid drugs and a relative quantitation study on mixtures of glucose and inositol.

**Figure 7:**
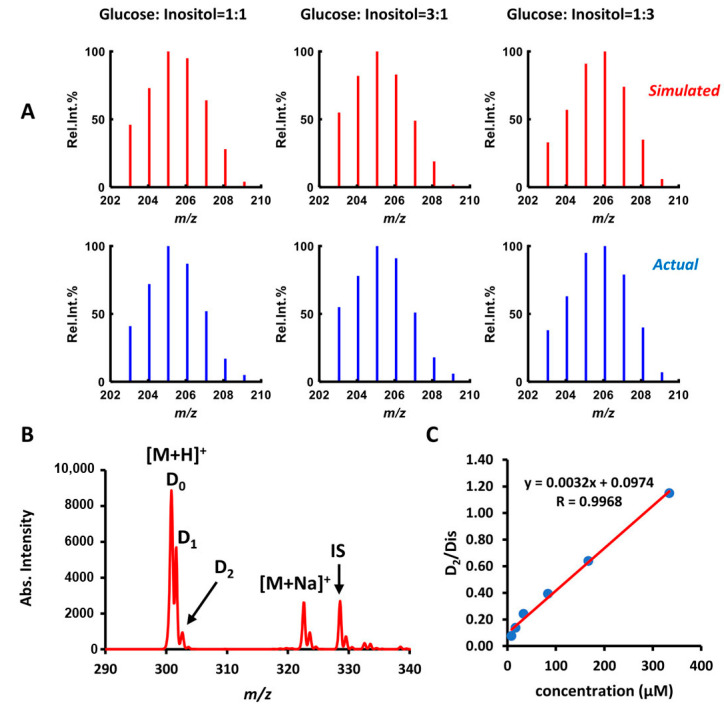
Evaluating the HDX–CPSI-MS in relative and absolute quantitation. (**A**) Simulated and actual HDX mass spectra collected from the mixed solution of glucose and inositol. (**B**) Representative HDX mass spectra of target codeine, and the internal standard (IS, 6-acetyl morphine). The interference compound, hydrocodone, was overlapped with codeine in peaks at *m*/*z* 300 (D_0_), and 301 (D_1_). However, the deuterium ion at *m*/*z* 302 (D_2_) only belongs to the target and was selected as the signature ion for quantitation; (**C**) Quantitation curve constructed by fitting the IS-calibrated response (D_2_/D_is_) versus codeine’s concentration.

First, given the fixed total concentration at 100 μM, solutions containing different molar ratios of glucose and inositol solution (2:2, 1:3, and 3:1) were prepared and tested by HDX-CPSI-MS. Given the hypothesis that two tested isobaric ions have very approximate ionization efficiencies, the absolute intensity of each deuterium peak (*D*_k_: k = 0–6) in a mixed HDX pattern was first simulated by a linear combination of two pure HDX patterns according to Formula (1), which is shown below. *N*_g_ and *N*_i_ denote the molar numbers for glucose and inositol. *I*g and *I*i denote the certain deuterium peak (*D*_k_: k = 0–6) intensity from pure glucose and pure inositol solutions, respectively. Then, the relative intensity of each deuterium peak can be normalized by the base peak according to Equation (2). *I*_BP_ denotes the absolute intensity of the base peak. A loss function in Equation (3) was proposed as a metric to evaluate the closeness between a simulation and an actual HDX pattern. Here “n” denotes the number of deuterium peaks. The “sim” and “obs” in Equation (3) refer to the simulated and observed intensities. As can be seen from Figure 7A, the simulated HDX patterns can be quite close to the actual ones. The scores of losses for three mixed samples of different molar ratios ranged from 2.8 to 5.3% (Supplementary Materials Table S2). This result indicates the feasibility of HDX-CPSI-MS for the relative quantitation of two isobaric ion, with the premise that they share a quite close ion efficiency.

(1) $$\;I_{abs}=\;N_{g}\times I_{g}+N_{i}\times I_{i}$$

(2) $$\;I_{rel\;}\left(\%\right)=\;100\times \frac{I_{abs}}{I_{BP}}\;$$

(3) $$Loss=\;\frac{1}{n+1}\;\sum_{i=0}^{k}|I_{sim}-I_{obs}|$$

We investigated another pair of isobaric ions (codeine, and hydrocodone). A series of codeine solutions (5, 10, 20, 50, 100, and 200 μg/mL) was spiked with a fixed concentration of hydrocodone (35 μg/mL), and an internal standard solution (6-acetylmorphine, 50 μg/mL) to construct samples tested by HDX–CPSI-MS. To rule out interference from hydrocodone, the specific deuterium peak from codeine (D_2_) was selected as the quantitative ion (Figure 7B). A quantitation curve was constructed by fitting the codeine molar concentration with calibrated responses based on the ratio of D_2_ ion versus the internal standard ion (D_is_). It was shown that the quantitation curve reflected an ideal linear relationship between the codeine’s concentration and its specific deuterated ion with a Pearson coefficient of 0.9968 (Figure 7C). It should be noted that this absolute quantitation was only suitable for one of the isobaric ions that has a unique deuterium signature. After obtaining the concentration of this isobaric ion, it is possible to estimate the concentration of the other one by the relative quantitation strategy we mentioned above. Please note that this analysis requires knowledge of the separate deuterium isotope shifts for each isobaric ion, and this can be regarded as a limitation of this technique.

To sum up, some of isomers or isobaric ions that are difficult to distinguish even by MS/MS experiments can be easily recognized by using HDX–CPSI-MS. Microdroplet HDX provides a cost-effective alternative for distinguishing between isobaric ions which can be complementary to CID-MS/MS fragmentation-based identification and ion-mobility-based separations. In terms of untargeted metabolomics, microdroplet HDX/mass spectrometry provides an extra dimension in that it is sensitive to active exchangeable sites of each MS peak which creates additional features in the profile. This behavior makes the global metabolic pattern more recognizable by multivariate analysis or machine learning and it also helps to find the underlying intricate differences. Additionally, it should be noted that an HDX pattern depends on not only the number of exchangeable sites and types of functional groups but also on the pH, percentage of D_2_O in the spraying solvent, etc. Therefore, for a fair comparison of two isobaric ions’ HDX patterns, the external conditions should be strictly controlled and made the same. Given the fixed external condition, particularly in pH, and D_2_O ratio, the day-to-day variation of a deuterium isotope’s relative abundance can be kept in RSD less than 15%. This level of reproducibility is usually sufficient to distinguish between different isobaric ions.

## 3. Methods

### 3.1. Reagents and Materials

All model compounds were purchased from Sigma-Aldrich (St. Louis, MO, USA), including metabolites (glucose and inositol), controlled narcotics (codeine, hydrocodone, 6-acetylmorphine, naloxone, morphine, and norcodeine), and isomers (para-/meta-/ortho-aminobenzoic acid). D_2_O (99.9 atom % D), methanol (99.9%), deionized ultra-filtered water, ammonium hydroxide, and glacial acetic acid (99.7%) were all obtained from Fisher Scientific. Polymethyl methacrylate (PMMA) was purchased from Titan Scientific Co. Ltd. (Shanghai, China), and multi-walled carbon nanotubes (MWCNT, ID 2–5 nM, OD < 8 nM, length 10–30 μM) were purchased from J&K Scientific Ltd. (Beijing, China).

### 3.2. Solution Preparation and Bio-Sample Collection

Stock solutions of glucose and inositol were prepared in ultrapure water with concentrations set as 20 μM. For each narcotics standard, a stock solution was constructed in methanol with a concentration of 20 μM. Urine samples of bladder cancer (BC) and healthy control (HC) volunteers were collected from the Veterans Affairs Palo Alto Healthcare System. In each case, consent was given in writing for samples acquired and followed the guidelines of the IRB (internal review board).

### 3.3. Droplet Spray Ionization

Conductive polymer spray ionization (CPSI) was employed as the ambient ionization method for the investigation of microdroplet HDX. For a CPSI experiment, the polymer substrate, which is made of PMMA and MWCNT, was cut into a triangular shape (8.0 mm wide and 10.0 mm high). Details of its step-by-step fabrication protocol can be found elsewhere [28]. For PSI and CPSI analysis, the biological fluid or compound solution (3 μL) was micropipetted onto the triangular tip of the substrate and fully dried to form a spot for analysis. A positive 4.5 kV high voltage was applied by a metal alligator clip onto the substrate that was positioned 13 mm in front of the mass spectrometer inlet. Then, methanol–H_2_O or methanol–D_2_O (5 μL, 7:3, *v*/*v*) was drop-wise loaded onto the conductive polymer tip. Driven by the strong electric field, charged microdroplets leave the conductive polymer substrate and head for the entrance to the mass spectrometer. During this process, transient HDX was taking place as captured and recorded in the mass spectrum.

### 3.4. Data Acquisition and Processing

An LTQ Orbitrap mass spectrometer (Thermo Fisher, San Jose, CA, USA) was employed for HDX data acquisition. For untargeted metabolic profiling, two duplicates of saliva or urine (3 μL) were first loaded onto tips of two paper or conductive polymer substrates to form dried fluid spots (DFSs), respectively. After the high voltage was powered on, a droplet of methanol–H_2_O or methanol–D_2_O (7:3, *v*/*v*, 5 μL) was spiked onto the DFS to trigger the metabolic profiling without or with HDX. Mass spectra within the range of *m*/*z* 50–1000 under both polarities were recorded. The MS capillary temperature was set at 275 °C. The tube lens and capillary voltage were set at 35 V and 110 V, respectively. The mass resolution in this study was set at 120,000. The number of microscans was set at 1 and the maximum injection time was set at 100 μs. The automatic gain control (AGC) function in this study was turned off.

The Xcalibur software (Thermo Fisher Scientific, San Jose, CA, USA) was employed for generating the average mass spectrum for each sample. Each spectrum was saved into a txt file for further processing. The in-built functions and self-programmed scripts under the MATLAB 2021 (Mathworks, Natick, MA, USA) were used for accessing txt files, total ion current normalization, and searching for deuterated peaks. SIMCA-P (Umetrics, Umea, Sweden) was used for multivariate analysis principal component analysis (PCA).

### 3.5. Isobaric Ion Discrimination and Assignment

Any unknown ion was first searched through HMDB (http://hmdb.ca/, accessed on 22 October 2021) and Metlin (https://metlin.scripps.edu, accessed on 22 October 2021) with the mass tolerance set at 5.0 ppm. Given the metabolites found in the libraries, we narrowed down the possibilities using knowledge of the specimens. The collision-induced dissociation (CID)-MS/MS experiment was also implemented to match the CID fragmentation pattern either with given standards or recorded MS/MS spectra in the database. For those metabolites that shared the same parent structure or very close MS/MS pattern, the proposed microdroplet HDX strategy was employed for further investigation and intricate discrimination. When the *m*/*z* mass shift of an ion falls into 1.0063 ± 0.0002, it can be considered as one deuterium replacement.

## 4. Conclusions

The ultrafast HDX process for active protons can be readily captured by AIMS using microdroplet HDX mass spectrometry. This new technique provides structural information about the number of exchangeable sites from a metabolite. Microdroplet HDX mass spectrometry is demonstrated to be an easy tool for isobaric ion discrimination and can be practically useful in a scenario in which metabolite candidates share the same parent structure and similar MS/MS patterns. In untargeted metabolomic studies, the microdroplet HDX-based metabolic profiling creates a new dimension for increasing pattern differences and facilitating the direct observation of inter-group pattern difference without statistical analysis, thus showing its promise as an additional tool for metabolite biomarker discovery.

## Data Availability

Data available in a publicly accessible repository. The data presented in this study are openly available in Open Science Framework (OSF, https://osf.io/q8ky5/, accessed on 22 October 2021).

## Supplementary Materials

The following are available online at www.mdpi.com/article/10.3390/metabo11110728/s1, The supporting information provides additional information on the MS/MS spectra of opioid drugs, as well as list of isobaric ions that were frequently detected in biological fluids and successfully distinguished by HDX-CPSI-MS: Figure S1: CID-MS/MS spectra of codeine, and hydrocodone, Figure S2: CID-MS/MS spectra of 6-acetyl morphine, and naloxone, Figure S3: CID-MS/MS spectra of morphine, and norcodeine, Tabls S1: List of isobaric ions that were frequently detected in saliva or serum and successfully distinguished by HDX-CPSI-MS, Table S2: List of isobaric ions that were frequently detected in biological fluids and successfully distinguished by HDX-CPSI-MS.

## References

[1] Feider C.L., Krieger A.C., DeHoog R.J., Eberlin L.S. (2019). Ambient Ionization Mass Spectrometry: Recent Developments and Applications. Anal. Chem..

[2] Takats Z., Wiseman J.M., Gologan B., Cooks R.G. (2004). Mass Spectrometry Sampling Under Ambient Conditions with Desorption Electrospray Ionization. Science.

[3] Wang Q., Bhattarai M., Zhao P., Alnsour T., Held M., Faik A., Chen H. (2020). Fast and Sensitive Detection of Oligosaccharides Using Desalting Paper Spray Mass Spectrometry (DPS-MS). J. Am. Soc. Mass Spectrom..

[4] Yang Q., Wang H., Maas J.D., Chappell W.J., Manicke N.E., Cooks R.G., Ouyang Z. (2012). Paper spray ionization devices for direct, biomedical analysis using mass spectrometry. Int. J. Mass Spectrom..

[5] Su Y., Wang H., Liu J., Wei P., Cooks R.G., Ouyang Z. (2013). Quantitative paper spray mass spectrometry analysis of drugs of abuse. Analyst.

[6] Li Z., Li Y., Zhan L., Meng L., Huang X., Wang T., Li Y., Nie Z. (2021). Point-of-Care Test Paper for Exhaled Breath Aldehyde Analysis via Mass Spectrometry. Anal. Chem..

[7] Chiu K.-Y., Wang Q., Gunawardena H.P., Held M., Faik A., Chen H. (2021). Desalting paper spay mass spectrometry (DPS-MS) for rapid detection of glycans and glycoconjugates. Int. J. Mass Spectrom..

[8] Rossini E.L., Kulyk D.S., Ansu-Gyeabourh E., Sahraeian T., Pezza H.R., Badu-Tawiah A.K. (2020). Direct Analysis of Doping Agents in Raw Urine Using Hydrophobic Paper Spray Mass Spectrometry. J. Am. Soc. Mass Spectrom..

[9] Yao Y.-N., Di D., Yuan Z.-C., Wu L., Hu B. (2020). Schirmer Paper Noninvasive Microsampling for Direct Mass Spectrometry Analysis of Human Tears. Anal. Chem..

[10] Wang T., Zheng Y., Wang X., Austin D.E., Zhang Z. (2017). Sub-ppt Mass Spectrometric Detection of Therapeutic Drugs in Complex Biological Matrixes Using Polystyrene-Microsphere-Coated Paper Spray. Anal. Chem..

[11] Chamberlain C., Hatch M., Garrett T. (2021). Extracellular Vesicle Analysis by Paper Spray Ionization Mass Spectrometry. Metabolites.

[12] Song X., Yang X., Narayanan R., Shankar V., Ethiraj S., Wang X., Duan N., Ni Y.-H., Hu Q., Zare R.N. (2020). Oral squamous cell carcinoma diagnosed from saliva metabolic profiling. Proc. Natl. Acad. Sci. USA.

[13] Li C., Li K., Xu X., Qi W., Hu X., Jin P. (2021). A pilot study for colorectal carcinoma screening by instant metabolomic profiles using conductive polymer spray ionization mass spectrometry. Biochim. Biophys. Acta (BBA)-Mol. Basis Dis..

[14] Mendes T.P.P., Pereira I., De Lima L.A.S., Morais C., Neves A.C., Martin F.L., Lima K.M.G., Vaz B.G. (2020). Paper Spray Ionization Mass Spectrometry as a Potential Tool for Early Diagnosis of Cervical Cancer. J. Am. Soc. Mass Spectrom..

[15] Mahmud I., Pinto F.G., Rubio V.Y., Lee B., Pavlovich C.P., Perera R.J., Garrett T.J. (2021). Rapid Diagnosis of Prostate Cancer Disease Progression Using Paper Spray Ionization Mass Spectrometry. Anal. Chem..

[16] Huang Y.-C., Chung H.-H., Dutkiewicz E.P., Chen C.-L., Hsieh H.-Y., Chen B.-R., Wang M.-Y., Hsu C.-C. (2019). Predicting Breast Cancer by Paper Spray Ion Mobility Spectrometry Mass Spectrometry and Machine Learning. Anal. Chem..

[17] Burnum-Johnson K.E., Zheng X., Dodds J.N., Ash J., Fourches D., Nicora C.D., Wendler J.P., Metz T.O., Waters K.M., Jansson J.K. (2019). Ion mobility spectrometry and the omics: Distinguishing isomers, molecular classes and contaminant ions in complex samples. TrAC Trends Anal. Chem..

[18] Maleki H., Maurer M.M., Ronaghi N., Valentine S.J. (2017). Ion Mobility, Hydrogen/Deuterium Exchange, and Isotope Scrambling: Tools to Aid Compound Identification in ‘Omics Mixtures. Anal. Chem..

[19] Uppal S.S., Beasley S.E., Scian M., Guttman M. (2017). Gas-Phase Hydrogen/Deuterium Exchange for Distinguishing Isomeric Carbohydrate Ions. Anal. Chem..

[20] Kelly K., Bell S., Maleki H., Valentine S.J. (2019). Synthetic Small Molecule Characterization and Isomer Discrimination Using Gas-Phase Hydrogen–Deuterium Exchange IMS-MS. Anal. Chem..

[21] Wu F., Huang Y., Yu F., Li Z.H., Ding C.-F. (2020). Effect of Transition-Metal Ions on the Conformation of Encephalin Investigated by Hydrogen/Deuterium Exchange and Theoretical Calculations. J. Phys. Chem. B.

[22] Trabjerg E., Nazari Z.E., Rand K.D. (2018). Conformational analysis of complex protein states by hydrogen/deuterium exchange mass spectrometry (HDX-MS): Challenges and emerging solutions. TrAC Trends Anal. Chem..

[23] Majuta S.N., Li C., Jayasundara K., Karanji A.K., Attanayake K., Ranganathan N., Li P., Valentine S.J. (2019). Rapid Solution-Phase Hydrogen/Deuterium Exchange for Metabolite Compound Identification. J. Am. Soc. Mass Spectrom..

[24] Narayanan R., Song X., Chen H., Zare R.N. (2020). Teflon Spray Ionization Mass Spectrometry. J. Am. Soc. Mass Spectrom..

[25] Dulay M.T., Boeser C.R., Walker K.L., Feider C., Zare R.N. (2021). Polymer Substrate with Surface Solvent Reservoir for Polymer-Spray Mass Spectrometric Analysis of Hydrophilic Drugs. Talanta Open.

[26] Dulay M.T., Zare R.N. (2017). Polymer-spray mass spectrometric detection and quantitation of hydrophilic compounds and some narcotics. Rapid Commun. Mass Spectrom..

[27] Song X., Chen H., Zare R.N. (2021). Coulometry--assisted quantitation in spray ionization mass spectrometry. J. Mass Spectrom..

[28] Song X., Chen H., Zare R.N. (2018). Conductive Polymer Spray Ionization Mass Spectrometry for Biofluid Analysis. Anal. Chem..

[29] Song X., Zang Q., Zare R.N. (2021). Hydrogen–Deuterium Exchange Desorption Electrospray Ionization Mass Spectrometry Visualizes an Acidic Tumor Microenvironment. Anal. Chem..

[30] Hamuro Y. (2021). Tutorial: Chemistry of Hydrogen/Deuterium Exchange Mass Spectrometry. J. Am. Soc. Mass Spectrom..

[31] Liyanage O.T., Brantley M.R., Calixte E.I., Solouki T., Shuford K.L., Gallagher E.S. (2019). Characterization of Electrospray Ionization (ESI) Parameters on In-ESI Hydrogen/Deuterium Exchange of Carbohydrate-Metal Ion Adducts. J. Am. Soc. Mass Spectrom..

[32] Kostyukevich Y., Acter T., Zherebker A., Ahmed A., Kim S., Nikolaev E. (2018). Hydrogen/deuterium exchange in mass spectrometry. Mass. Spec. Rev..

