# Supplementary material for: Distinguishing between Isobaric Ions Using Microdroplet Hydrogen–Deuterium Exchange Mass Spectrometry
Source: Metabolites. 2021 Oct 23;11(11):728. doi: 10.3390/metabo11110728 (PMC8625015; doi:10.3390/metabo11110728)
Supplement: Supplementary file 1 [file metabolites-11-00728-s001.zip › metabolites-1425336-supplementary.pdf]

# SUPPORTING INFORMATION

## Distinguishing Between Isobaric Ions Using Microdroplet Hydrogen-Deuterium Exchange Mass Spectrometry

Xiaowei Song[1,2], Jia Li[2], Mohammad Mofidfar[1], Richard N. Zare[1*]

1. Department of Chemistry, Stanford University, Stanford, CA, USA. 94305
2. Department of Chemistry, Fudan University, Shanghai, China. 200438

**Correspondence**

Richard N. Zare: rnz@stanford.edu

**Table of Contents**


| Figure/Table | Legend | Pages |
|---|---|---|
| Figure S1 | CID-MS/MS spectra of codeine, and hydrocodone. | S2 |
| Figure S2 | CID-MS/MS spectra of 6-acetyl morphine, and naloxona. | S3 |
| Figure S3 | CID-MS/MS spectra of morphine, and norcodeine. | S4 |
| Table S1 | List of isobaric ions that were frequently detected in saliva or serum and successfully distinguished by HDX-CPSI-MS. | S5 |
| Table S2 | List of isobaric ions that were frequently detected in biological fluids and successfully distinguished by HDX-CPSI-MS. | S6 |

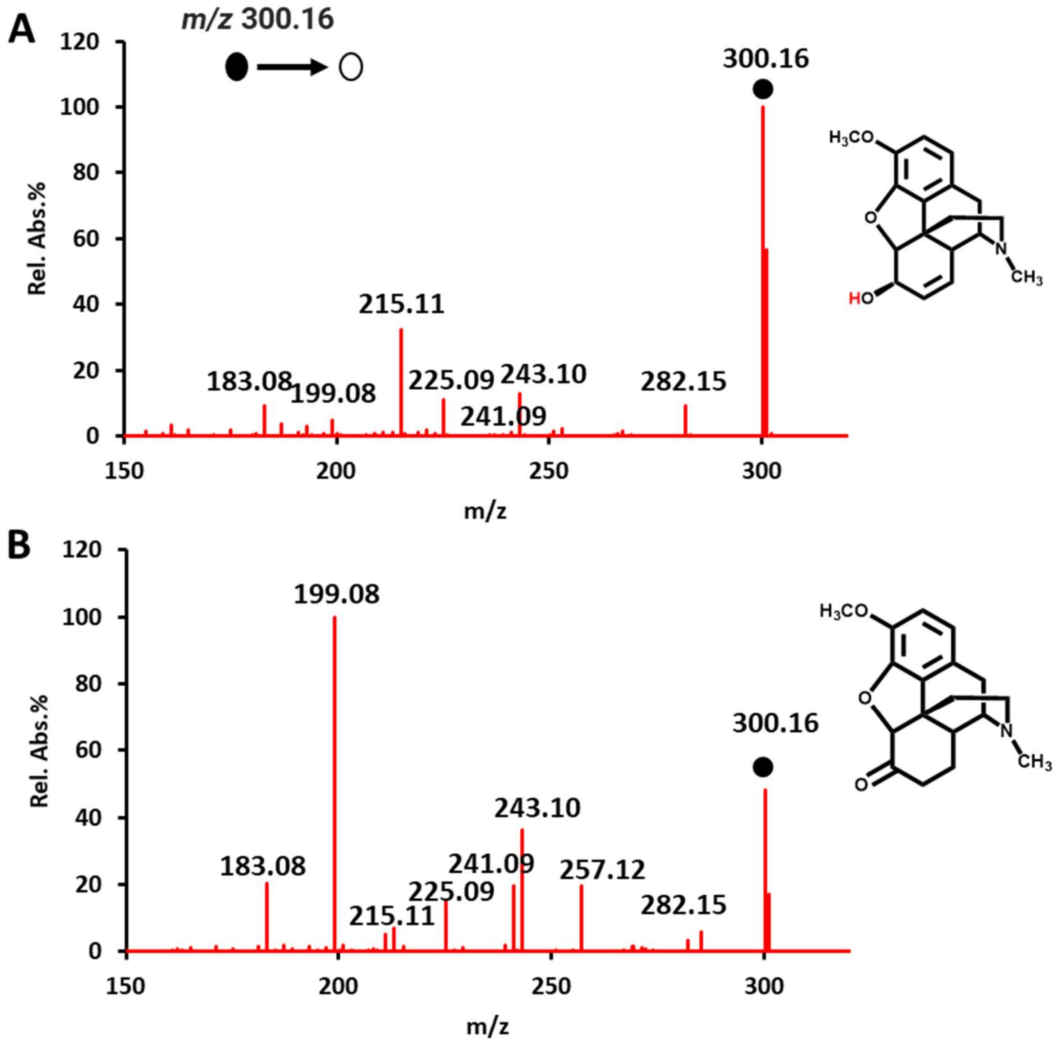


**Figure S1. CID-MS/MS spectra of (A) codeine and (B) hydrocodone. The black dot indicates the precursor ion.**

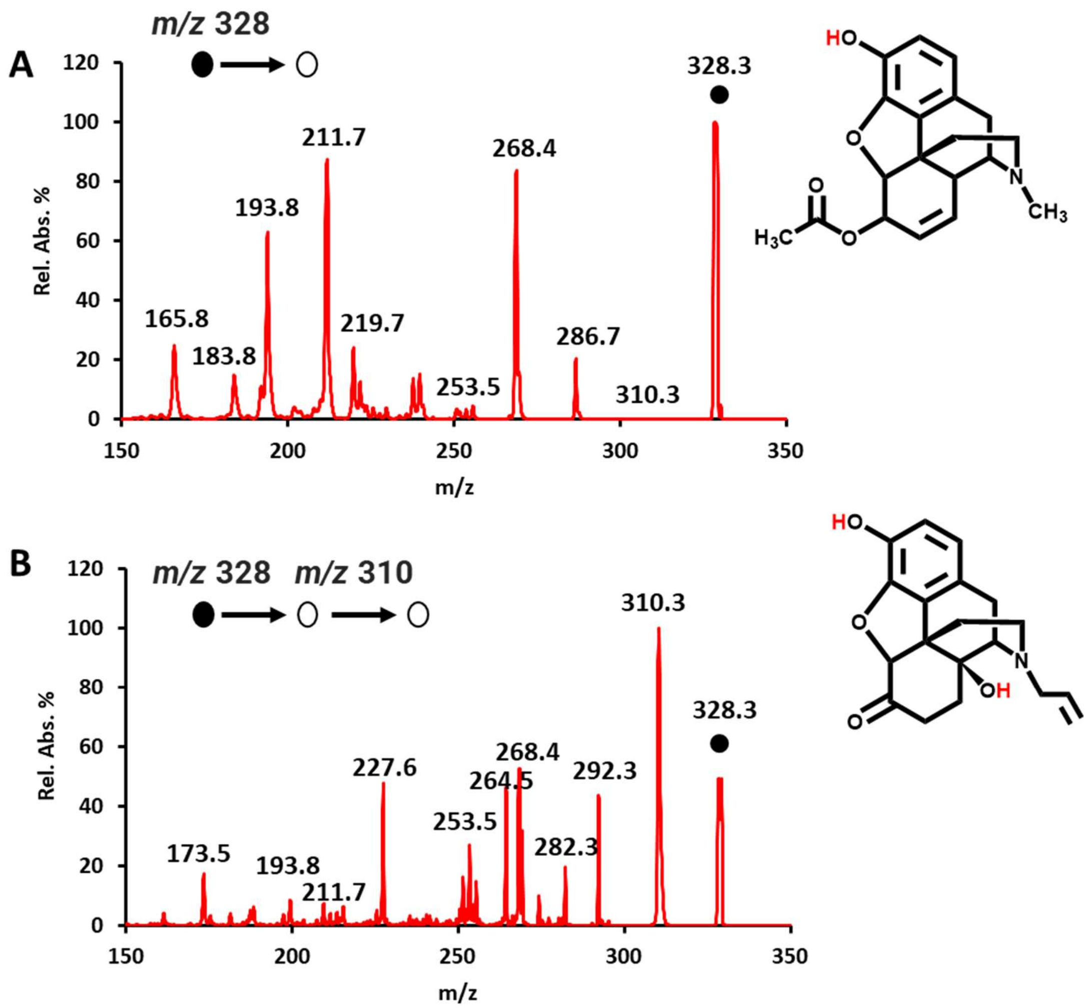


**Figure S2. CID-MS/MS spectra for (A) 6-acetyl morphine and CID-MS3 experiment for (B) naloxone. The black dot indicates the precursor ion.**

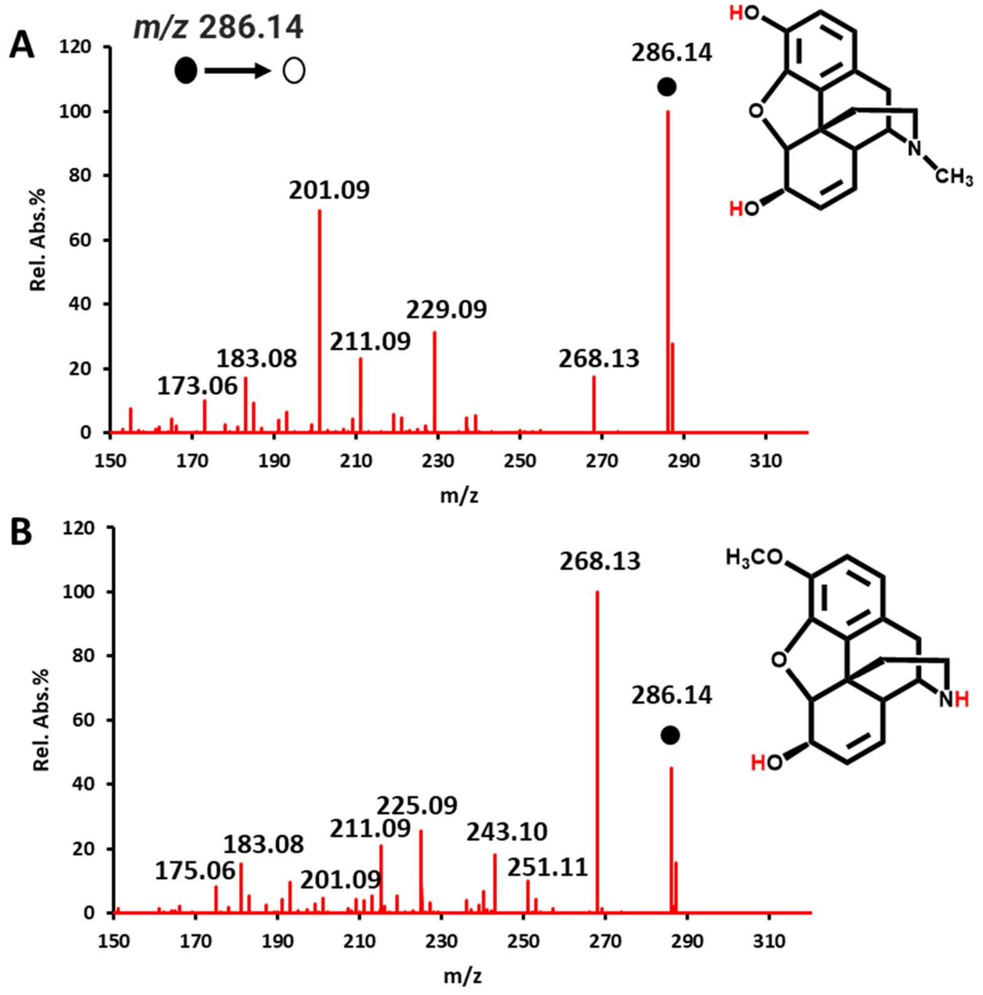


**Figure S3. CID-MS/MS spectra of (A) morphine and (B) norcodeine. The black dot indicates the precursor ion.**

## Table S1. Isobaric ions that were frequently detected in saliva or serum and successfully distinguished by HDX-CPSI-MS.

| No | Exact *m/z* | Adduct Ions | Formulae | Metabolites | Number of Exchangeable proton sites | Base peaks |
|---|---|---|---|---|---|---|
| 1 | 118.0862 | $[M+H]^+$ | $C_5H_{11}NO_2$ | Betaine | 1 | $D_0$ |
| | | | | 5-Aminopentanoic acid | 3 | $D_1$ |
| | | | | Valine | 3 | $D_0$ |
| 2 | 141.0789 | $[M+K]^+$ | $C_5H_{14}N_2$ | Cadaverine | 4 | $D_2$ |
| | | | | N-methyl putrescine | 3 | $D_2$ |
| 3 | 146.0459 | $[M-H]^-$ | $C_5H_9NO_4$ | Glutamate | 3 | D2 |
| | | | | O-acetyl serine | 2 | $D_0$ |
| | | | | N-acetyl serine | 2 | $D_1$ |
| 4 | 280.0904 | $[M+Na]^+$ | $C_{10}H_{15}N_3O_5$ | 5-Methylcytidine | 5 | $D_2$ |
| | | | | 3-methylcytidine | 4 | $D_2$ |
| | 280.0920 | $[M+H]^+$ | $C_8H_{20}NO_6P$ | glycerophosphocholine | 3 | $D_2$ |
| 5 | 112.0369 | $[M+Na]^+$ | $C_3H_7NO_2$ | Alanine | 3 | $D_1$ |
| | | | | Sarcosine | 2 | $D_1$ |
| 6 | 126.0520 | $[M+Na]^+$ | $C_4H_9NO_2$ | aminobutyric acid | 3 | $D_1$ |
| | | | | N, N-dimethylglycine | 1 | $D_0$ |
| 7 | 137.0706 | $[M+H]^+$ | $C_7H_8N_2O$ | 2-aminobenzamide | 4 | $D_1$ |
| | | | | N-methylnicotinamide | 1 | $D_0$ |

**Table S2. The relative intensities in HDX patterns acquired from samples of glucose (G) and inositol (I) at different molar ratios.**

| *m/z* | G:I Simulated HDX Patterns | | | G:I Observed HDX Patterns | | |
|---|---|---|---|---|---|---|
| | 1:1 | 3:1 | 1:3 | 1:1 | 3:1 | 1:3 |
| 203.05 | 46 | 55 | 33 | 41 | 55 | 38 |
| 204.06 | 73 | 82 | 57 | 72 | 78 | 63 |
| 205.07 | 100 | 100 | 91 | 100 | 100 | 95 |
| 206.08 | 95 | 83 | 100 | 87 | 91 | 100 |
| 207.09 | 64 | 49 | 74 | 52 | 51 | 79 |
| 208.10 | 28 | 19 | 35 | 17 | 18 | 40 |
| 209.11 | 4 | 2 | 6 | 5 | 6 | 7 |
